# The Queen Adelaide Fund of the Hanwell Lunatic Asylum

**Published:** 1841-01

**Authors:** J. Conolly, C. A. Tulk

**Affiliations:** Chairman of the Visiting Magistrates of the County Lunatic Asylum


### THE QUEEN ADELAIDE FUND OF THE HANWELL LUNATIC ASYLUM.

This is a charity attached to the great Middlesex County Asylum, and was instituted for the relief of destitute patients at the time of their recovery. Its nature and object, and the great benefits it is calculated to afford to the unhappy subjects of it, are set forth so admirably in the following letter, that we are gratified in being able to give it extensive circulation in our pages; and are not without hopes that some of our readers may be induced by its perusal to contribute to the charity or to induce others to do so. We the more readily republish the letter that it contains some striking though brief illustrations of the causes and miserable consequences of insanity among the poorer classes:

"Dear Sir, *Hanwell; February* 3, 1840.

"In the Report which I had lately the honour to lay before the visiting magistrates of the asylum, it was observed that the extreme poverty of some of the patients, and the certainty that on being discharged from the asylum they would return to a miserable home, and be exposed to many causes most likely to produce a relapse, sometimes occasioned hesitation respecting the propriety of sending them away, after they were sufficiently restored to reason to make their restoration to the ordinary habits of life desirable. It was added, that the benefit arising, in many instances, from the Adelaide fund was so great and so evident as to make its limited extent deeply to be regretted.

"Having almost daily opportunities of seeing the good done by this excellent fund, and, I am sorry to add, of seeing cases to which its limited assistance cannot be extended, and in which such charitable help is greatly required, I trust you will permit me to lay before you a few particulars illustrative of these circumstances.

"It very often happens that insanity makes its first advances slowly; manifesting itself by eccentric and irregular conduct, fits of illness, of dissipation, or of extravagance long before it is clearly recognized. These first symptoms of the disorder, inconvenient in every rank of life, are ruinous to a family dependent on the daily labour of a husband and a father.

"By their frequent recurrence or by their long continuance, every comfort is successively sacrificed, and every article of furniture and dress that can possibly be spared becomes pledged for a little money to meet the daily necessities of the patient, of the helpless children, and of his almost broken-hearted wife; who is long unable to account for the demoralization which is bringing ruin upon the whole family. At length the malady becomes too plain to be mistaken, and the poor lunatic, after being delayed a short time in the workhouse, and a longer time in some cheap private lunatic house, is brought to the asylum; and his wife and children are taken care of by the parish. In a few months, perhaps, the poor man recovers. He then begins daily to represent to us the deserted state of his family and his anxiety to help them; and becoming at length quite well he is discharged. When he takes off the asylum dress, he clothes himself in the ragged worn out garments which have been kept for him at the workhouse; often fancying that his best clothes have been stolen, forgetting how he parted from them; and going away in some degree of irritation on this account. He then either goes to the workhouse or into the poorest lodging in the lowest retreats of destitution. He finds more difficulty in obtaining work than he expected. His having been insane operates against the success of his efforts to be employed; he is pressed with daily wants which were not felt or thought of in the asylum; and in short, exposed, immediately after his recovery, to every probable cause of relapse.

"In many instances the patient's malady has been of longer duration. When he leaves the asylum he finds that his friends are dead or have almost forgotten him; and he learns all at once the troubles with which those for whom he feels affection have long been struggling. Many of these patients first become insane after long contention with all these evils; and were worn and harassed by various wretchedness until they lost their reason. When we turn any of these unfortunate persons out of the gate pennyless, we at once expose them to a repetition of the causes of their first attack of madness or of melancholy.

"The instances are numerous in which poor widows are admitted, distracted by the failure of some humble business, to the carrying on of which they were unequal after their husband's death. Affliction and sorrow in these cases commonly produce the most marked examples of a profound and speechless melancholy, from which the recovery is slowly effected if effected at all. No means of assisting or of confirming recovery are so likely to be efficacious as being able to hold out the promise of a little aid toward the re-establishment of some business by which these patients, when restored to some degree of cheerfulness, may look forward to being enabled to live honestly. I believe the benevolent persons who have superintended the formation and distribution of the Adelaide Fund have witnessed not a few most affecting cases of this kind; in which, also, the aid derived from the fund became the blessed instrument of regained prosperity.

"Of 244 female patients, of whom the station or occupation are mentioned in a table appended to the Michaelmas Report of the Asylum, 125 were domestic servants. These poor women, when recovered, are of course seldom able at once to find places. Their affliction has seldom been concealed from those who know them or with whom they formerly lived. Unless they can go to their parents the workhouse is their only resource. It often happens that their parents are extremely poor, and ill able to support any additional burthen. A small donation in these cases gives the destitute girl a kind of welcome to her home, and enables her to go to it with confidence and cheerfulness, and she exerts herself and does well. Without such help and her mind still weak, her condition would often have become very lamentable.

"We have also, at all times, among our patients, some in whom, after the severer symptoms of their malady have disappeared, a slight disorder or impairment of the mind remains, or a certain eccentricity of manner, or a disposition to excitement when contradicted or not judiciously managed. These patients under the guardianship of the various officers and servants of the asylum who are familiar with their character, are most industrious labourers, or at least most serviceable assistants, and, whilst pleased and engaged in different employments of more or less consequence, are placed in circumstances extremely favorable to permanent cure. But these patients, conscious that they are useful, and sometimes even overrating their services, would often become discontented and refuse to leave their wards, or to be in any way active without the encouraging hope of assistance when discharged. In these cases, the prospect of pecuniary aid, although they know it can only be trifling, becomes auxiliary to the perfect cure of the patient, promotes satisfaction, stimulates activity, and at the same time contributes to restore the powers of body and of mind.

"We are often placed in a peculiarly painful situation as respects country patients, who have no parish to return to. The miserable fate that inevitably awaits some of them causes us, indeed, occasionally to keep them week after week because we know that they have, whilst with us, needful food and clothing and shelter, and being unable when they leave us to furnish them either with clothes or with money, we cannot bear to see them turned out of the asylum in rags, and without a farthing and without a friend. In these circumstances we cannot always refrain from extending a little help to them, for which we should be glad to have fuller means and authority. A suit of clothes, costing about twelve or fifteen shillings, and a sum of money not exceeding one or two sovereigns, when the recovered patient has a long journey before him, are absolutely necessary to prevent their being regarded as vagrants or even perishing on the road. I am convinced, both by what has been communicated to me and by my own observation, that assistance to this extent has, in some cases, perhaps in many, saved a poor man or woman from total abandonment and despair, has been the means of restoring them to their distant families, and of securing their subsequent well-doing.

"Constantly occupied in the treatment of a class of maladies, the history of which reveals so much physical and moral weakness, and circumstances of such complicated and terrible distress, we feel that we have done but a part of our duty when we have merely contributed to restore the sanity of the mind, and anxiously look for the completion of our efforts to any possible means of restoring the victim of insanity to a position in society, promising some tranquillity of heart and a consolatory hope of moderate prosperity. Except the opportunities afforded to us by the Adelaide Fund, to the benevolent founders of which we can never feel sufficiently grateful, we have no means of effecting these most desirable ends. The number of patients in the asylum when that fund was established did not exceed 300, it now amounts to 834, and preparation has lately been made for the reception of 100 more. If, therefore, the fund could be so brought before the attention of the County of Middlesex, and of benevolent persons in other parts of the kingdom, as to show them how important its benefits are to the largest lunatic establishment in the kingdom, built solely for the relief of paupers, it might possibly be so increased as to enable the magistrates and the officers of the asylum to extend its charitable succour to many for whom, at present, there is unfortunately no provision of any kind, no hope, and no resource.

"I remain, very sincerely, dear Sir,
"Your obedient servant,
"J. CONOLLY.

"To C. A. Tulk, Esq., Chairman of the
"Visiting Magistrates of the County Lunatic Asylum."

